# A Diethylene Glycol-Regulated Aqueous Electrolyte for Zinc-Ion Hybrid Capacitors Operating from −40 to 60 °C

**DOI:** 10.3390/molecules31173001

**Published:** 2026-08-27

**Authors:** Wentao Li, Ying Liu, Xingchao Wang, Xiaohan Jiang, Lingyang Liu

**Affiliations:** Shandong Provincial Key Laboratory of Chemical Energy Storage and Novel Cell Technology, School of Chemistry and Chemical Engineering & School of Energy Science and Technology, Liaocheng University, Liaocheng 252059, China; liwentao418chem@163.com (W.L.); wangxingchao2018@163.com (X.W.); xhjiang_2021@163.com (X.J.)

**Keywords:** aqueous zinc-ion hybrid capacitor, diethylene glycol, wide-temperature electrolyte, zinc plating/stripping, electrolyte regulation

## Abstract

Aqueous zinc-ion hybrid capacitors (ZIHCs) have attracted considerable attention for safe and sustainable energy storage; however, their practical application under variable temperature conditions remains challenging due to electrolyte freezing at low temperatures, accelerated water-related side reactions at elevated temperatures, and the instability of zinc electrode reactions. Herein, a diethylene glycol (DG)-based aqueous electrolyte was developed to improve the wide-temperature electrochemical performance of ZIHCs. The effects of different DG concentrations on electrolyte properties, molecular interactions, and electrochemical behavior were systematically investigated. The incorporation of an appropriate amount of DG regulates the hydrogen bond interactions among water molecules, thereby reducing water-related side reactions and improving zinc reversibility. Among the investigated electrolytes, the optimized DW80 electrolyte achieves balanced performance in terms of safety, low-temperature tolerance, and electrochemical stability. Benefiting from the optimized electrolyte composition, Zn||Zn symmetric cells exhibit stable long-term cycling performance, and the assembled ZIHCs demonstrate reliable operation over a wide temperature range from −40 to 60 °C. This work highlights the importance of optimizing cosolvent content and provides a simple strategy for improving the temperature adaptability of aqueous zinc-based energy storage systems.

## 1. Introduction

Aqueous zinc-ion hybrid capacitors (ZIHCs) have attracted considerable interest as promising energy storage devices owing to their intrinsic safety, low cost, environmental compatibility, and the high theoretical capacity of zinc metal [1,2,3,4,5,6]. Compared with conventional lithium-based systems, aqueous zinc-based devices utilize abundant and non-flammable electrolytes, offering potential advantages for large-scale energy storage applications. However, the practical performance of ZIHCs is strongly dependent on the physicochemical properties of aqueous electrolytes, especially under variable temperature conditions. The narrow operating temperature range of conventional aqueous electrolytes remains a major limitation to their reliable operation in harsh environments [7,8,9].

At low temperatures, the formation of ice crystals and decreased ionic mobility can severely restrict ion transport and deteriorate electrochemical performance. Meanwhile, elevated temperatures accelerate water evaporation, parasitic reactions, and zinc electrode corrosion, leading to poor cycling stability [10,11,12,13,14]. Various strategies have been developed to improve the temperature tolerance of aqueous zinc electrolytes, including increasing salt concentration, introducing organic solvents, and constructing gel-based electrolytes [15,16,17,18,19]. Although these approaches can effectively improve either low-temperature resistance or high-temperature stability, achieving a balance between electrolyte stability, ionic conductivity, and reversible zinc deposition remains challenging.

Cosolvent-based electrolyte regulation provides a simple approach to modify water structures and improve the temperature adaptability of aqueous zinc systems [20,21,22,23,24,25]. Organic molecules containing hydroxyl or ether groups can interact with water molecules through hydrogen bonding, thereby adjusting water activity and influencing electrolyte phase behavior [26,27,28,29,30,31]. Diethylene glycol (DG), a low-cost and commercially available ether alcohol solvent, possesses multiple oxygen-containing functional groups and a high boiling point, making it a potential candidate for regulating aqueous electrolyte properties. However, the effects of DG incorporation on hydrogen bond interactions, Zn^2+^ solvation environments, and the electrochemical behavior of zinc-based hybrid capacitors require further investigation.

Herein, a DG-regulated aqueous electrolyte was developed for wide-temperature ZIHCs. The influence of DG incorporation on the electrolyte structure was systematically investigated through spectroscopic characterization and molecular dynamics simulations. The results reveal that DG modifies the hydrogen bond interactions among water molecules and participates in the Zn^2+^ solvation environment, contributing to suppressed water-related side reactions and improved zinc reversibility. As a result, the DG-based ZIHCs exhibit stable electrochemical performance from −40 to 60 °C, demonstrating the effectiveness of DG as a simple cosolvent for improving the temperature adaptability of aqueous zinc-ion energy storage systems.

## 2. Results

### 2.1. Hydrogen Bond Network Regulation and Wide-Temperature Adaptability of DG-Based Electrolytes

**Figure 1:**
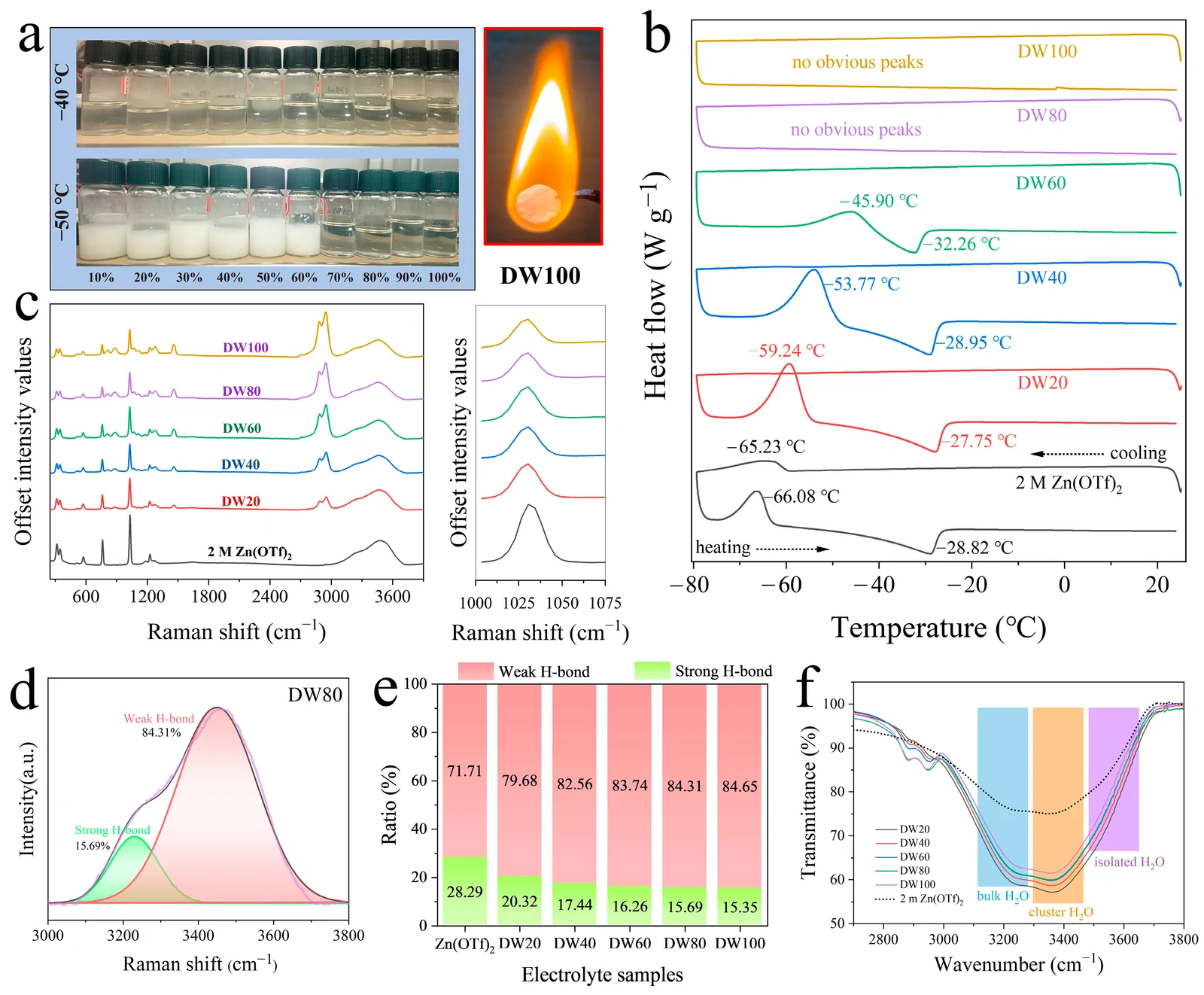
(**a**) Digital photographs of Zn(OTf)_2_-based electrolytes with different DG/H_2_O volume ratios in the mixed solvent under −40 °C and −50 °C. (**b**) DSC cooling and heating curves of 2 M Zn(OTf)_2_ and DG-containing electrolytes. (**c**) Raman spectra of DG-containing electrolytes revealing the evolution of water molecular structures. (**d**) Deconvolution of the O–H stretching region into strongly hydrogen-bonded and weakly hydrogen-bonded water species in DW80 electrolyte. The purple curves represent the original experimental data, while the black curves correspond to the fitted results. (**e**) Quantitative analysis of the hydrogen-bond configurations with different DG concentrations. (**f**) FTIR spectra of pristine aqueous and DG-regulated electrolytes demonstrating the interaction between DG molecules and water molecules.

The introduction of organic cosolvents provides an effective strategy to regulate the physicochemical properties of aqueous electrolytes and expand their temperature tolerance. In this work, diethylene glycol (DG), a low-cost ether alcohol solvent containing abundant hydroxyl and ether oxygen groups, was selected as a hydrogen bond-regulating cosolvent to construct Zn(OTf)_2_/DG/H_2_O hybrid electrolytes. As shown in Table S1, a series of electrolytes with different DG/H_2_O volume ratios were prepared, denoted as DWx, where x represents the volume percentage of DG relative to water in the mixed solvent (e.g., DW20 corresponds to a DG/H_2_O volume ratio of 20:100). The effects of DG incorporation on the phase behavior and molecular interactions of the electrolytes were systematically investigated. The temperature-dependent phase behavior of DG-based electrolytes was first evaluated by visual observation under subzero conditions. As is well known, the aqueous Zn(OTf)_2_ electrolyte rapidly loses transparency and exhibits obvious freezing behavior at low temperatures. As shown in Figure 1a, all DG-containing electrolytes remain transparent and fluid at −40 °C, indicating a markedly improved low-temperature tolerance imparted by the cosolvent. Upon further cooling to −50 °C, a distinct compositional dependence emerges: electrolytes with DG/H_2_O volume ratios from 10% to 60% gradually solidify, whereas those with ratios of 70% and above maintain a homogeneous liquid state. However, when the temperature is lowered to −55 °C, all tested electrolytes eventually freeze (Figure S1). This progressive freezing behavior underscores the critical role of DG content in tuning the anti-freezing capability, which originates from the strong hydrogen-bonding interactions between DG molecules and water. Specifically, the hydroxyl (–OH) and ether (–O–) groups of DG form abundant hydrogen bonds with water molecules, effectively disrupting the ordered water network that serves as the structural basis for ice nucleation. Combustion tests further reveal a clear correlation between DG content and electrolyte flammability. A bare separator burns completely upon direct flame exposure. By contrast, separators loaded with ~100 µL of DW20, DW40, DW60, or DW80 exhibit no ignition or visible shrinkage, indicating that the water-rich composition effectively suppresses combustion. In particular, DW100 exhibits obvious combustion behavior due to the high content of organic solvent, whereas electrolytes with moderate DG contents maintain better safety characteristics. Nevertheless, the overall non-flammability of the DG/H_2_O hybrid electrolytes remains a critical advantage for practical grid-scale energy storage applications, where intrinsic safety is paramount.

Differential scanning calorimetry (DSC, −80 to 25 °C) was used to quantify the freezing behavior of the electrolytes (Figure 1b and Table S2). On cooling, only the baseline electrolyte shows a crystallization exotherm (Tc ≈ −65 °C), whereas all DG-containing electrolytes remain free of crystallization, indicating that DG suppresses ice crystallization. The lack of an exotherm could also arise from supercooling. More importantly, on heating, DW20–DW60 exhibit clear melting endotherms with melting points Tm of −27.75 (ΔH = 31.69 J g^−1^), −28.95 (ΔH = 22.00 J g^−1^), and −32.26 °C (ΔH = 13.09 J g^−1^), respectively, meaning that these electrolytes are crystalline below ~−30 °C and therefore cannot remain liquid at −40 °C. In sharp contrast, DW80 shows no melting peak (ΔH ≈ 0.06 J g^−1^), and DW100 also shows only a negligible melting event (ΔH = 0.28 J g^−1^). These quantitative data demonstrate that DW80 and DW100 remain essentially amorphous or liquid across the entire temperature window, replacing the previous visual observation and directly supporting the wide-temperature operation of the optimized DW80 electrolyte. The difference relative to Figure 1a arises largely from the variation in solution loading amounts, as DSC testing uses only a small mass (<10 mg) of electrolyte. In addition, the cooling process can induce supercooling effects.

The ionic conductivity of all electrolytes at 25, 0, −20, and −40 °C was measured using a conductivity meter in a programmable environmental chamber. The conductivity decreases monotonically with increasing DG content at every temperature and drops sharply at subzero temperatures (Table S3). Importantly, although the baseline shows the highest absolute conductivity, it freezes at −40 °C and therefore becomes non-functional; in contrast, DW80 retains a measurable 0.223 mS cm^−1^ at −40 °C while staying in the liquid state. Among the DG-containing electrolytes, DW100 exhibits the lowest conductivity (0.113 mS cm^−1^ at −40 °C), explaining its inferior subzero rate capability. Together with the DSC results, these data quantitatively corroborate the low-temperature behavior and support DW80 as the optimized composition that balances acceptable ionic transport with a non-freezing state across the −40–60 °C window. Subsequently, the viscosity of all electrolytes was measured at 25 and −20 °C using a rotational rheometer, and the results are reported in Table S4. As can be seen, the viscosity increases monotonically with the DG content and rises sharply as the temperature is lowered to −20 °C. At 25 °C, the viscosity increases from 3.37 ± 0.002 mPa·s (baseline) to 10.81 ± 0.012 mPa·s (DW80) and 24.13 ± 0.75 mPa·s (DW100); at −20 °C, it reaches 204.9 ± 1.2 mPa·s (baseline), 876.5 ± 2.30 mPa·s (DW80) and 1420.5 ± 6.91 mPa·s (DW100), the latter being the highest in the series. The high viscosity of DW100, especially at −20 °C, significantly impedes ion transport and raises the polarization penalty, which quantitatively explains its inferior subzero cycling performance. In contrast, DW80 retains a more favorable viscosity–conductivity balance: its viscosity is markedly lower than that of DW100 while it still remains liquid over the whole target window. Together with the ionic-conductivity data, this supports DW80 as the optimized composition that best reconciles low-temperature fluidity with stable electrochemical performance.

The molecular interaction between DG and water was further revealed by Raman spectroscopy. As displayed in Figure 1c, the characteristic vibration bands associated with water structures gradually evolve with increasing DG concentration. The broad O–H stretching region provides direct information regarding different hydrogen bond states of water molecules [32,33,34,35,36]. Compared with the pristine Zn(OTf)_2_ aqueous electrolyte, DG-containing electrolytes exhibit an increased contribution from weakly hydrogen-bonded water species, indicating the disruption of the highly interconnected water hydrogen bond network. In addition, the Raman spectra in the range of 1000–1075 cm^−1^ were further analyzed to investigate the interaction between Zn^2+^ and OTf^−^ anions. The characteristic band located around 1030 cm^−1^ is assigned to the symmetric stretching vibration of OTf^−^. With increasing DG content, the gradual shift in this vibration indicates the modification of the OTf^−^ coordination environment, suggesting that DG participates in regulating ion interactions and the Zn^2+^ solvation structure. As shown in Figure 1d and Figure S2, the O–H stretching region (3000 cm^−1^ to 3800 cm^−1^) can be deconvoluted into strongly hydrogen-bonded (3230 cm^−1^) and weakly hydrogen-bonded (3430 cm^−1^) water components. To quantitatively analyze the evolution of hydrogen bond configurations of water molecules in electrolytes, the relative proportions of strongly and weakly hydrogen-bonded water were summarized in Figure 1e. In the Zn(OTf)_2_ electrolyte, strongly hydrogen-bonded water accounts for 28.29%, while weakly hydrogen-bonded water accounts for 71.71%. With the gradual addition of DG (DW20, DW40, DW60, DW80, DW100), the proportion of strongly hydrogen-bonded water continuously declines from 20.32% to 15.35%, whereas the fraction of weakly hydrogen-bonded water monotonically rises from 79.68% to 84.65%. Notably, the DW80 electrolyte exhibits a markedly lower proportion of strong hydrogen bonding (15.69%), with weakly hydrogen-bonded water accounting for up to 84.31% of the hydrogen-bond environment. The O–H stretching region contains contributions from both water and the hydroxyl groups of DG, which overlap spectrally. To separate them without overdecomposition, we exploited the fact that DG is independently identifiable through its own fundamental vibrations lying outside the O–H region: DG exhibits characteristic C–O–C ether stretching at ~850 cm^−1^ and CH_2_ deformations near 1450 cm^−1^ that are absent in pure water and therefore serve as unambiguous internal fingerprint markers for DG. Because the DG hydroxyl itself contributes only a minor fraction to the total O–H envelope relative to the dominant bulk water signal, the evolution of the strongly vs. weakly hydrogen-bonded water ratio with DG content directly reflects DG-induced disruption/restructuring of the water hydrogen bond network rather than an artifact of the added DG hydroxyl. Such variation unambiguously verifies that DG effectively breaks the original dense hydrogen bond network among water molecules and restructures the intermolecular interactions of solvent water. The influence of DG on water molecular states was further confirmed by FTIR spectroscopy (Figure 1f and Figure S3). Compared with the Zn(OTf)_2_ aqueous electrolyte, DG-containing electrolytes display weakened absorption intensity in the characteristic region associated with bulk water, together with the emergence of more isolated water species [37,38,39,40]. These results suggest that DG molecules effectively interact with water molecules and reduce the fraction of freely existing water, thereby suppressing undesirable phase transitions at low temperatures and improving electrolyte stability. Collectively, these results demonstrate that DG serves as an effective molecular regulator for aqueous electrolytes by modulating the hydrogen bond network of water. The strong DG–H_2_O interactions weaken the continuous water network, inhibit ice crystal formation under low-temperature conditions, and provide the foundation for constructing wide-temperature aqueous zinc-based energy storage systems.

### 2.2. Investigation of DG-Induced Molecular Interactions and Zn^2+^ Solvation Environment

**Figure 2:**
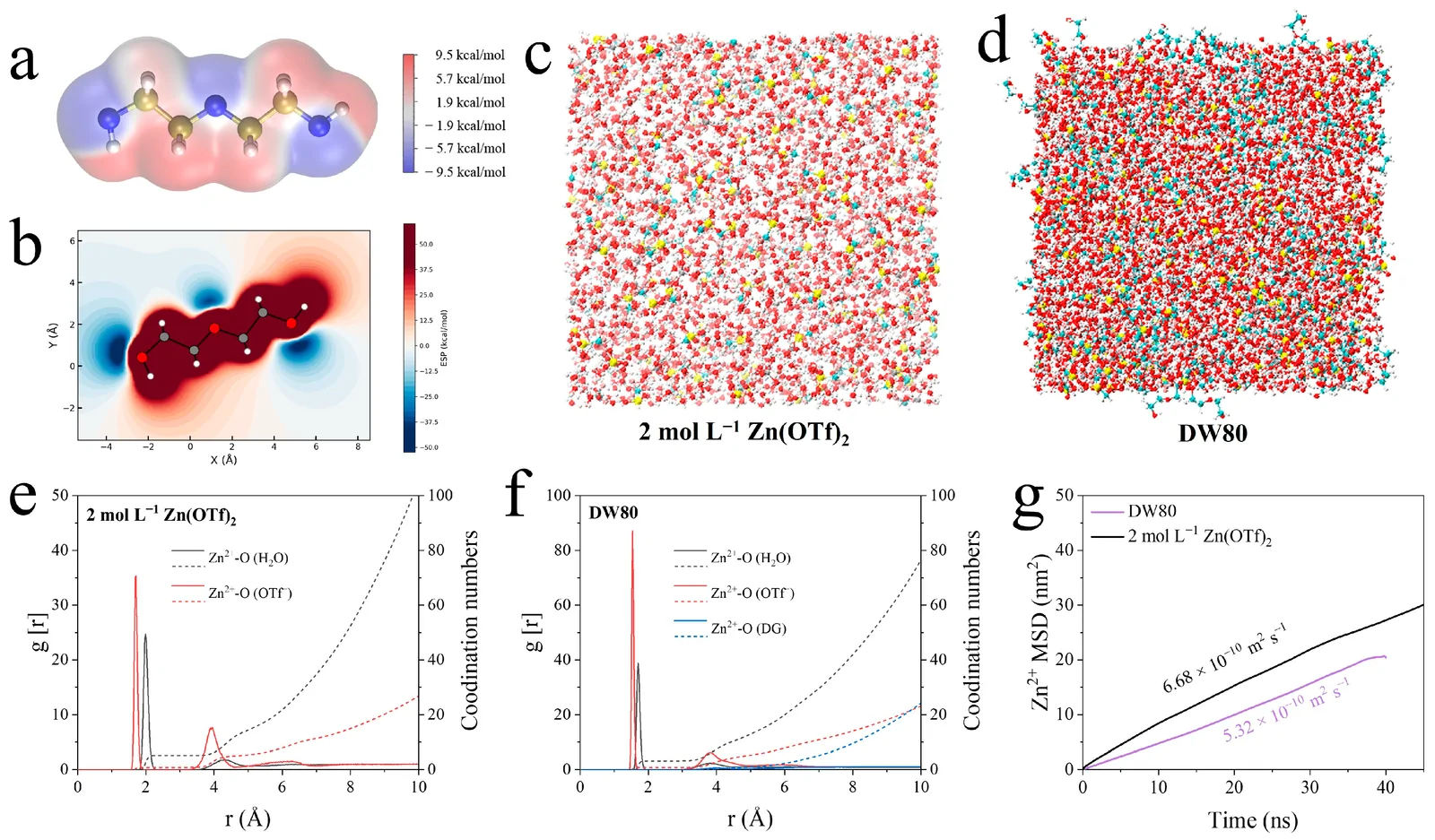
(**a**) Three-dimensional and (**b**) two-dimensional molecular electrostatic potential (ESP) distribution of DG molecules showing the polar oxygen-containing functional groups. (**c**,**d**) Representative snapshots of molecular dynamics simulations for pure Zn(OTf)_2_ aqueous electrolyte and DW80 electrolyte. Red, O atom; white, H atom; cyan, C atom; yellow, F atom; pink, S atom; gray, Zn atom. (**e**) Radial distribution functions (RDFs) and coordination numbers (CN) of Zn^2+^ with oxygen atoms from water molecules and anions in pure Zn(OTf)_2_ electrolyte. (**f**) RDFs and CN of Zn^2+^ with oxygen atoms from water molecules, DG molecules, and anions in DW80. (**g**) Mean-squared displacement (MSD) of the Zn^2+^ ions.

Following the hydrogen bond network regulation induced by DG, the influence of DG on the water molecules and local Zn^2+^ solvation environment was further investigated through molecular dynamics (MD) simulations. The molecular electrostatic potential (ESP) distribution of DG was first analyzed to understand its interaction capability with water molecules and Zn^2+^ ions. As shown in Figure 2a,b, the three-dimensional and two-dimensional distributions of DG molecules show that oxygen atoms in both hydroxyl and ether groups exhibit pronounced negative electrostatic regions, indicating strong hydrogen bond-accepting ability and coordination activity. These polar functional groups enable DG molecules to interact strongly with surrounding water molecules and participate in the construction of Zn^2+^ solvation structures [41,42,43]. The spatial distribution of DG molecules and Zn^2+^ ions was further visualized by MD simulation. As shown in Figure 2c,d, the incorporation of DG significantly modifies the molecular arrangement within the electrolyte system. Compared with the pristine aqueous electrolyte, DG molecules are uniformly distributed around Zn^2+^ ions and water molecules, forming a more heterogeneous solvation environment. This molecular rearrangement is beneficial for weakening the excessive interaction between Zn^2+^ and water molecules and reducing the participation of free water in interfacial reactions.

The radial distribution functions(RDF, *g*(*r*)) and corresponding coordination number (CN) curves of Zn^2+^ in the pristine 2 mol L^−1^ Zn(OTf)_2_ and DW80 electrolytes are displayed in Figure 2e and Figure 2f, respectively. For the baseline Zn(OTf)_2_ and DW80 electrolytes, obvious characteristic peaks of Zn^2+^-O (H_2_O) and Zn^2+^-O (H_2_O) (OTf^−^) can be observed, which originate from water molecules and triflate anions participating in the solvation shell of Zn^2+^. The Zn^2+^–O(H_2_O) RDF exhibits a sharp first coordination shell peak at r = 1.70 Å, with a coordination number of 3.20 (integrated to the first minimum at r = 2.14 Å). The Zn^2+^–O(OTf^−^) RDF shows a first peak at r = 1.54 Å, with a coordination number of 0.80 (integrated to the first minimum at r = 1.82 Å). Notably, the Zn^2+^–O(DG) RDF exhibits no discernible first-shell peak (*g*(*r*) < 1, coordination number ≈ 0), indicating that DG does not directly coordinate Zn^2+^ within the first shell. The first solvation shell of Zn^2+^ in DW80 is instead composed of ~3.2 H_2_O and ~0.8 OTf^−^. This is expected, as Zn^2+^ is a hard Lewis acid with a strong preference for the small, high-donor-number water molecules over the sterically demanding, weakly donating ether/hydroxyl oxygens of DG. Importantly, this reveals that DG functions not by entering the Zn^2+^ solvation shell, but by reducing water activity and reconstructing the hydrogen bond network, thereby suppressing ice crystallization and water-induced side reactions, which is consistent with the DSC results (no crystallization/melting for DW80) and Raman/FTIR results (altered water O–H environment). Because DG leaves the Zn^2+^ solvation shell essentially intact, the fast (de)solvation kinetics are preserved while interfacial stability is enhanced. Meanwhile, the mean squared displacement (MSD) curves of Zn^2+^ for the two systems are presented in Figure 2g. The diffusion coefficient of Zn^2+^ in pure 2 mol L^−1^ Zn(OTf)_2_ electrolyte is calculated to be 6.68 × 10^−10^ m^2^ s^−1^. Upon DG addition (DW80), the Zn^2+^ diffusion coefficient slightly decreases to 5.32 × 10^−10^ m^2^ s^−1^. The decrease in this value is likely attributed to the increase in electrolyte viscosity and the decrease in ionic conductivity. Such a modified solvation environment is anticipated to reduce active free water surrounding Zn^2+^, which helps suppress the parasitic hydrogen evolution reaction during battery cycling.

### 2.3. Electrochemical Stability of Zinc Electrodes in DG-Based Electrolytes

**Figure 3:**
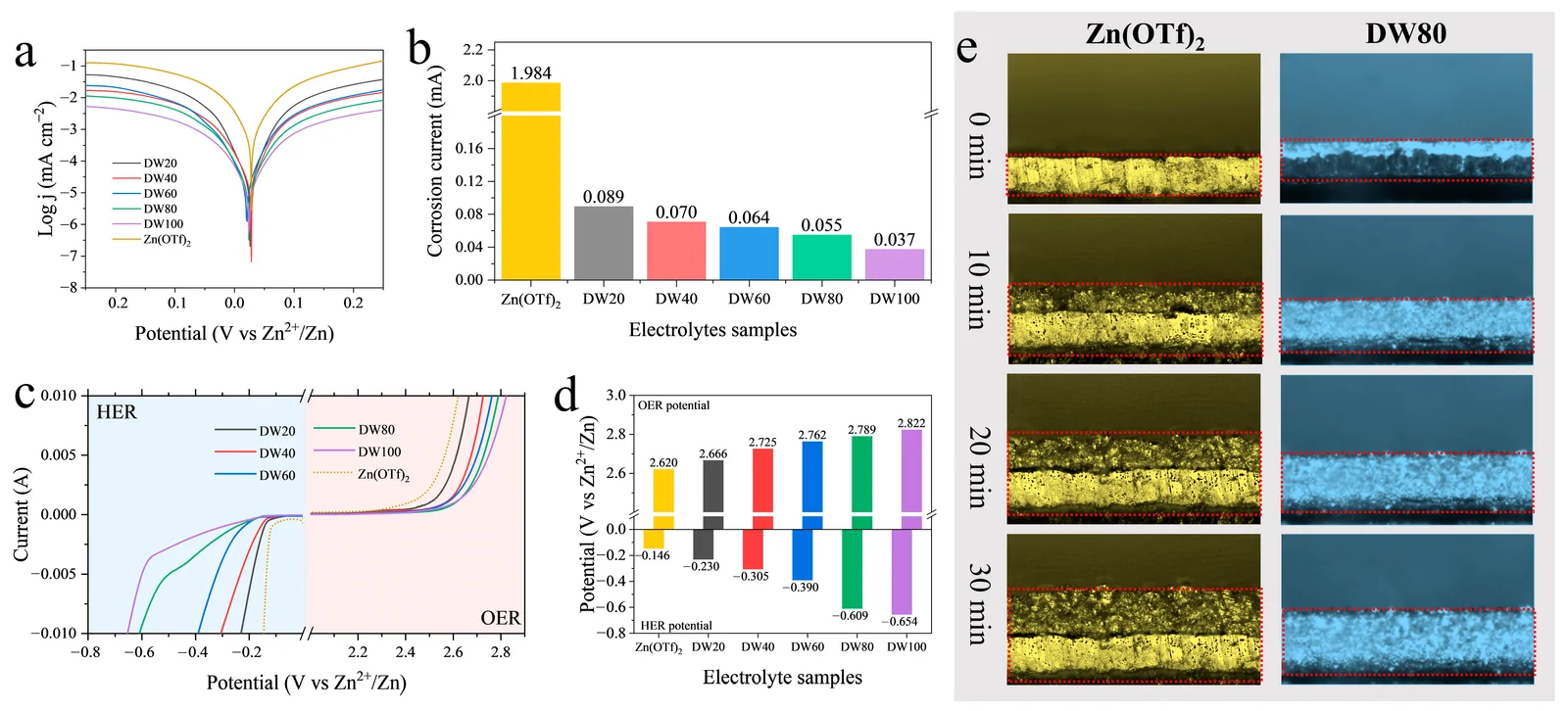
(**a**) Tafel curves of zinc electrodes in different electrolytes. (**b**) Corresponding corrosion current densities calculated from polarization measurements. (**c**) Linear sweep voltammetry (LSV) curves for evaluating hydrogen evolution reaction (HER) and oxygen evolution reaction (OER). (**d**) The summary of HER potentials and OER potentials of the different electrolytes. (**e**) In situ optical microscopy images showing the surface evolution of zinc electrodes during electrochemical polarization in 2 mol L^−1^ Zn(OTf)_2_ and DW80 electrolytes. The red dashed boxes mark the thickness of the zinc foil and the deposited zinc layer.

The regulation of water structure and the Zn^2+^ solvation environment by DG is expected to suppress parasitic reactions at the zinc/electrolyte interface. To clarify the influence of DG on zinc electrode stability, a series of electrochemical measurements were performed. Tafel polarization measurements (scan rate: 5 mV s^−1^) were initially conducted to evaluate the corrosion behavior of zinc electrodes in different electrolytes. As shown in Figure 3a,b, the corrosion current density of zinc in the pristine Zn(OTf)_2_ aqueous electrolyte is as high as 1.984 mA cm^−2^, indicating severe interfacial corrosion associated with abundant active water species. This value decreases monotonically with DG addition, falling to 0.089, 0.070, 0.064, 0.055, and 0.037 mA cm^−2^ for DW20, DW40, DW60, DW80, and DW100, respectively. The systematic suppression of corrosion current with increasing DG content can be attributed to the reduced water activity and regulated interfacial solvation environment, which collectively hinder the electrochemical dissolution of zinc [44,45].

Linear sweep voltammetry (LSV, scan rate: 5 mV s^−1^) measurements were performed to evaluate the electrochemical stability window of different electrolytes, as displayed in Figure 3c,d. For the pristine Zn(OTf)_2_ electrolyte, the HER onset potential is −0.146 V vs. Zn^2+^/Zn, while the OER onset potential reaches 2.620 V vs. Zn^2+^/Zn, delivering a narrow electrochemical stability window. With the continuous increase in DG content from DW20 to DW100, the HER onset potential gradually shifts negatively from −0.230 V to −0.654 V vs. Zn^2+^/Zn, and the OER onset potential progressively moves positively from 2.666 V to 2.822 V vs. Zn^2+^/Zn. The gradual negative shift in the HER potential shows that DG effectively suppresses the parasitic hydrogen evolution reaction and alleviates Zn metal corrosion induced by free water. Meanwhile, the positively elevated OER potential reveals suppressed oxygen evolution. Benefiting from the dual inhibition of HER and OER, the electrolyte with a DG additive achieves a substantially widened electrochemical stability window. Among all tested systems, the DW100 electrolyte exhibits the lowest HER onset potential (−0.654 V) and highest OER onset potential (2.822 V), demonstrating improved electrochemical stability. Such improved anodic and cathodic stability originates from the reconstruction of the water hydrogen bond network after DG incorporation, which reduces the amount of reactive free water in the electrolyte [46,47]. This electrochemical stability improvement is consistent with the above Raman and MD simulation results, wherein DG restructures hydrogen bond interactions and the Zn^2+^ solvation environment to consume active free water.

To directly observe the evolution of zinc surfaces during electrochemical operation, in situ optical microscopy was employed. As shown in Figure 3e, the zinc electrode in the Zn(OTf)_2_ aqueous electrolyte rapidly develops an uneven and rough surface accompanied by abundant by-product formation. In contrast, the DW80 electrolyte maintains a much smoother zinc surface with significantly reduced interfacial degradation during prolonged polarization. This observation confirms that DG-derived regulation of the electrolyte environment facilitates uniform zinc deposition and suppresses irreversible side reactions.

**Figure 4:**
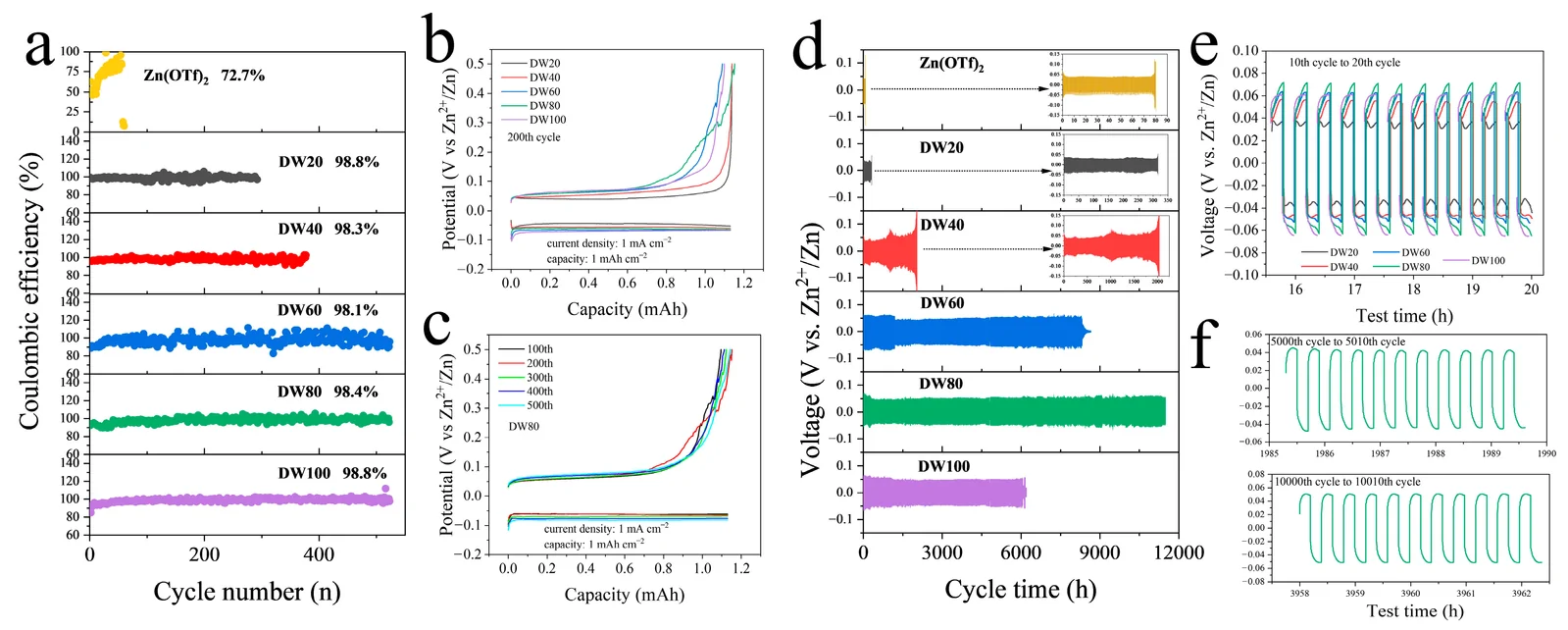
(**a**) Coulombic efficiency profiles of Zn plating/stripping in different electrolytes using Zn||Cu half-cells (current density of 1 mA cm^−2^ with a capacity of 1 mAh cm^−2^). (**b**) Galvanostatic plating/stripping profiles of Zn||Cu cells assembled with various electrolytes at 200th cycle. (**c**) The voltage profiles of Zn||Cu cells assembled with DW80 electrolyte at 100th, 200th, 300th, 400th and 500th cycles. (**d**) Long-term galvanostatic cycling voltage curves of Zn||Zn symmetric cells with various electrolytes (current density of 0.25 mA cm^−2^ with a capacity of 0.05 mAh cm^−2^). (**e**) Enlarged voltage profiles of the 10th–20th cycles for all electrolytes. (**f**) Magnified galvanostatic curves of DW80 electrolyte at 5000th to 5010th cycles and 10,000th to 10,010th cycles.

The reversibility and cycling stability of Zn plating/stripping in various DG-modified electrolytes were systematically evaluated using Zn||Cu half-cells and Zn||Zn symmetric cells (current density of 0.25 mA cm^−2^ with a capacity of 0.05 mAh cm^−2^) [48,49,50]. Figure 4a presents the Coulombic efficiency (CE) curves of Zn deposition/dissolution in Zn||Cu half-cells (current density of 1 mA cm^−2^ with a capacity of 1 mAh cm^−2^). The bare Zn(OTf)_2_ electrolyte delivers an unsatisfactory average CE of only 72.7% and suffers severe fluctuations during cycling, which originate from rampant hydrogen evolution, Zn corrosion, and uncontrolled dendrite formation. After introducing DG, all modified electrolytes achieve remarkably boosted average CE values exceeding 98%. Specifically, DW20, DW40, DW60, DW80, and DW100 exhibit average CE values of 98.8%, 98.3%, 98.1%, 98.4%, and 98.8%, respectively. Galvanostatic charge–discharge profiles at the 200th cycle from Zn||Cu cells are displayed in Figure 4b. Compared with other DG-containing electrolytes, DW60 and DW80 exhibit smaller voltage hysteresis during Zn plating/stripping, implying accelerated Zn^2+^ transfer kinetics and milder interfacial polarization. Figure 4c further records the voltage profiles of the DW80 electrolyte at different cycles (100th–500th). The voltage hysteresis remains nearly unchanged with prolonged cycling, demonstrating excellent interfacial stability of the DW80 electrolyte. In addition, the post-cycling SEM images of Zn electrodes harvested from three independent Zn||Cu cells based on the baseline and DW80 electrolytes after galvanostatic cycling were added. In the baseline electrolyte (Figure S4a), the cycled Zn surface is rough and non-uniform, exhibiting pronounced protrusions mossy or dendrite-like deposits. In contrast, the DW80 electrolyte (Figure S4b) yields a markedly flatter, denser, and more uniform Zn deposition with negligible dendritic features. The smoother, dendrite-suppressed morphology of Zn cycled in DW80 demonstrates that the DG-based electrolyte guides uniform Zn^2+^ plating/stripping and effectively inhibits dendrite growth and the associated parasitic side reactions.

Long-term galvanostatic cycling tests of Zn||Zn symmetric cells were performed to evaluate the reversibility and stability of Zn plating/stripping in various electrolytes, and the voltage–time profiles are displayed in Figure 4d. For the pristine Zn(OTf)_2_ electrolyte, the symmetric cell only maintains stable operation for ~80 h. Severe voltage distortion and large polarization fluctuations emerge rapidly, originating from vigorous hydrogen evolution, Zn corrosion, and uncontrolled dendrite growth induced by abundant free water. Benefiting from the introduction of DG, all modified electrolytes exhibit greatly prolonged cycling lifespans and stabilized voltage hysteresis. The cell with the DW20 electrolyte can steadily cycle for approximately 316 h. The DW40-based symmetric cell achieves a cycling duration of around 2000 h. Remarkably, the DW60 electrolyte enables the Zn||Zn cell to sustain ultra-long stable cycling over 8600 h with flat voltage curves and negligible polarization variation, demonstrating superior cycling stability. In comparison, cells utilizing DW80 and DW100 electrolytes run stably for ~11,500 h and ~6200 h, respectively. Figure 4e compares the voltage profiles of the 10th–20th cycles for all electrolytes. DW60 and DW80 display lower and more stable voltage hysteresis than other formulations at the initial stage. Figure 4f shows the magnified voltage curves of the DW80 electrolyte at different cycling periods (5000th–5010th and 10,000th–10,010th cycles). The well-preserved, smooth voltage profiles after long-term cycling confirm stable Zn deposition/stripping behavior without obvious polarization aggravation. To verify the stability of the Zn||Zn symmetric cells under high current density, we repeated the Zn||Zn cycling for the baseline and DW80 electrolytes at 1 mA cm^−2^ and 1 mAh cm^−2^ using three independent cells per electrolyte (Figure S5). As can be seen, Zn||Zn symmetric cells with 2 M Zn(OTf)_2_ failed after 68 h, 52 h, and 65 h, respectively. In contrast, three Zn||Zn symmetric cells with DW80 cycled steadily for over 110 h, demonstrating the superior interfacial stability of DW80. The comprehensive voltage hysteresis comparison across the three independent cells was also obtained. As expected, DW80 exhibits a somewhat larger voltage hysteresis than the baseline electrolyte (~61.8 mV for DW80 vs. ~35.3 mV for the baseline after 20 cycles). This larger polarization is a bulk transport trade-off inherent to the high-DG formulation and is quantitatively consistent with the reduced ionic conductivity of DW80.

These results confirm that DG effectively restrains free-water-triggered parasitic reactions, suppresses Zn anode corrosion, homogenizes Zn deposition, and significantly improves the cycling durability of metallic Zn anodes. The optimized solvation structure and reconstructed hydrogen bond network account for the enhanced stability in DG-containing electrolytes, among which DW80 presents the best performance. Collectively, the above results demonstrate that DG plays a critical role in stabilizing the zinc/electrolyte interface. By reducing free water activity, regulating Zn^2+^ solvation structures, and suppressing water-related side reactions, DG promotes uniform zinc deposition and enhances the reversibility of zinc electrodes. These interfacial improvements provide the foundation for constructing high-performance aqueous zinc-ion hybrid capacitors under wide-temperature conditions.

### 2.4. Wide-Temperature Electrochemical Performance of DG-Based ZIHCs

**Figure 5:**
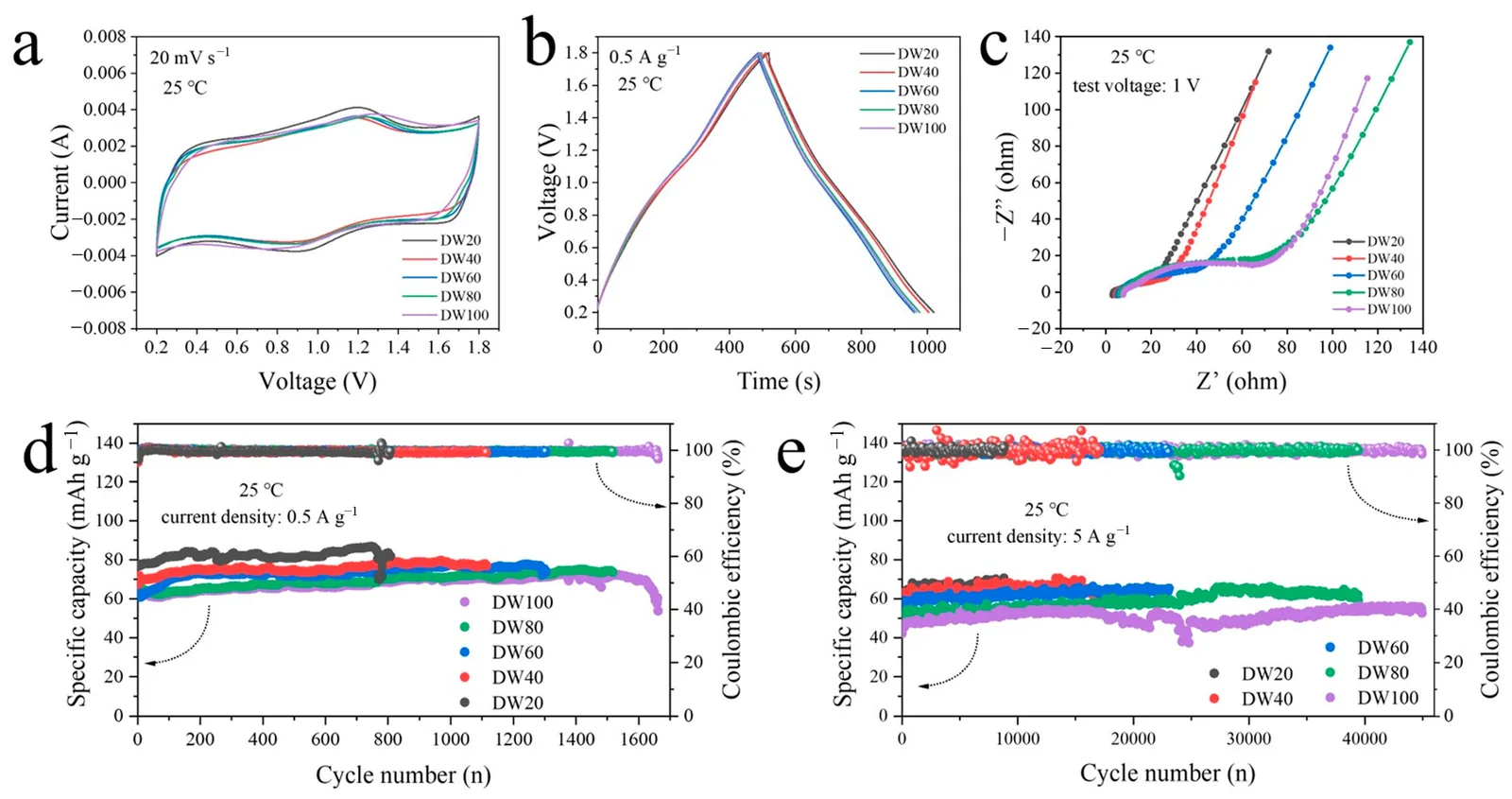
(**a**) Cyclic voltammetry (CV) curves of ZIHCs using different electrolytes at various scan rates. (**b**) Galvanostatic charge–discharge (GCD) profiles at different current densities. (**c**) Nyquist plots of ZIHCs measured by electrochemical impedance spectroscopy. Long-term cycling performance and Coulombic efficiency of ZIHCs (**d**) at low current density of 0.5 A g^−1^ and (**e**) at high current density of 5 A g^−1^.

To demonstrate the practical applicability of DG-regulated electrolytes, aqueous zinc-ion hybrid capacitors (ZIHCs) were assembled and evaluated under various temperature conditions. Before investigating extreme temperature operation, the electrochemical behavior of ZIHCs at room temperature was first examined. As shown in Figure 5a–c and Figures S6–S8, all DG-containing electrolytes exhibit comparable electrochemical characteristics, including similar cyclic voltammetry (CV) profiles, galvanostatic charge/discharge (GCD) curves, and impedance responses, suggesting that the introduction of DG does not compromise the fundamental electrochemical kinetics of the ZIHCs. It is worth noting that the addition of DG slightly affects the charge transfer speed, resulting in a slight decrease in specific capacity.

The long-term cycling stability of ZIHCs assembled with various electrolytes was evaluated at 25 °C under low and high current densities. Figure 5d displays the long-term cycling profiles at a current density of 0.5 A g^−1^. The ZIHCs with the DW20 electrolyte deliver stable cycling for around 800 cycles. Further increasing the DG content significantly extends the cycling lifespan. The DW40, DW60, and DW80 systems can steadily cycle for approximately 1110, 1300, and 1510 cycles, respectively. Impressively, the ZIHCs using the DW100 electrolyte achieve optimal cycling stability and maintain effective operation for up to 1600 cycles with mild capacity decay. All DG-modified electrolytes exhibit much slower capacity fading compared with the low-DG concentration group, demonstrating that DG can suppress parasitic reactions and stabilize the electrode-electrolyte interface under a moderate current density. Figure 5e presents ultra-long cycling tests conducted at a high current density of 5 A g^−1^. The DW20 electrolyte suffers severe capacity attenuation and fluctuating Coulombic efficiency and fails rapidly after roughly 8800 cycles. Benefiting from the optimized DG concentration, the DW40- and DW60-based cells can sustain stable cycling for nearly 15,000 and 23,000 cycles. The DW80 electrolyte enables the ZIHCs to realize superior long-cycle durability, operating smoothly for more than 40,000 cycles. The DW100 system also achieves outstanding cycling stability over 45,000 cycles with high coulombic efficiency. Such remarkable cycling performance under high-rate conditions highlights the merits of DG, which reconstructs the hydrogen bond network of THE aqueous electrolyte and modulates the Zn^2+^ solvation structure, reduces active free water, inhibits HER and zinc anode corrosion, and stabilizes reversible zinc deposition/stripping. Consequently, the full ZIHC cells achieve suppressed capacity decay and improved cycling lifespan, especially under high-current operation.

**Figure 6:**
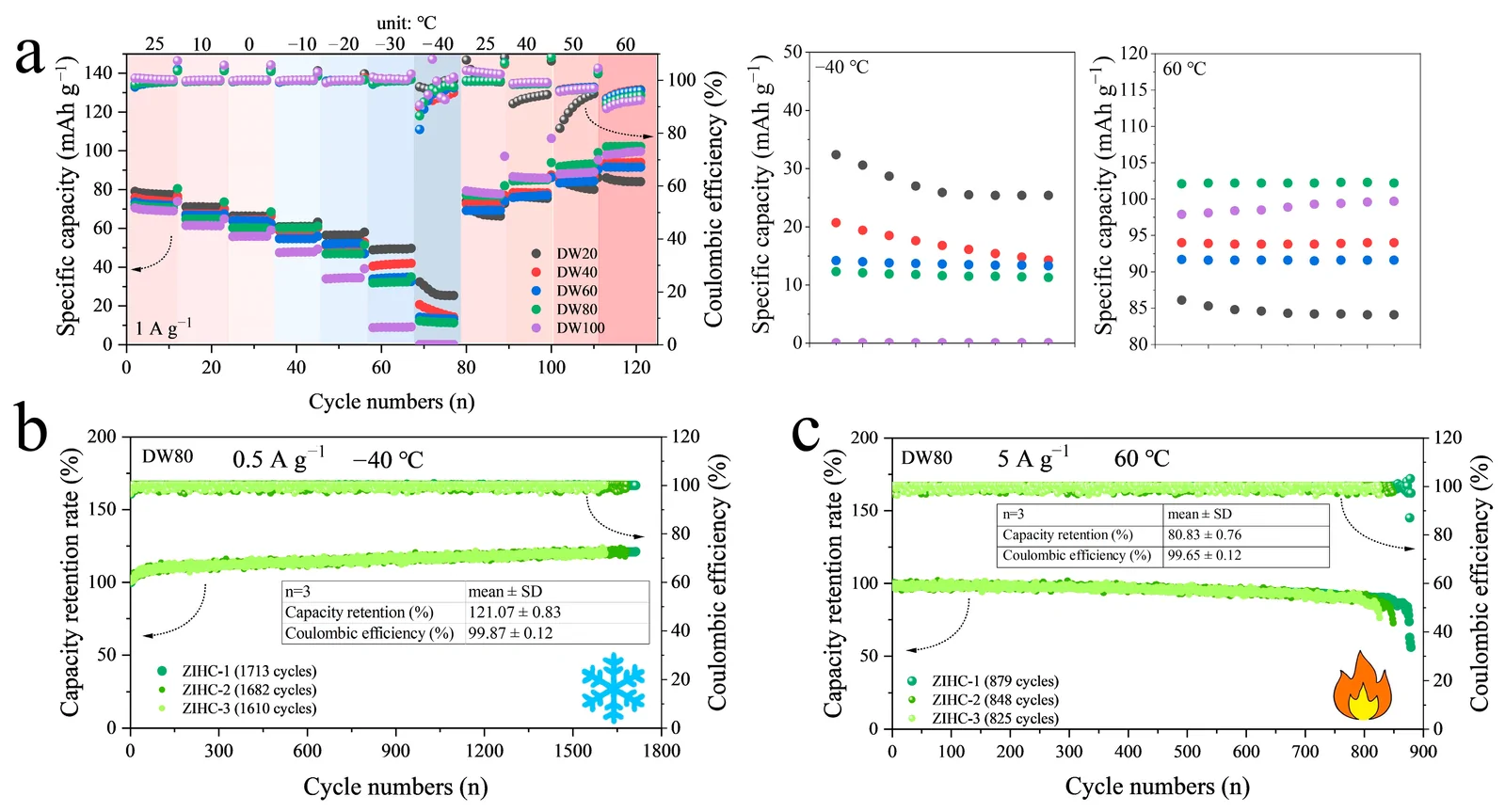
(**a**) Continuous cycling at a current density of 1.0 A g^−1^ of ZIHCs based on DW20, DW40, DW60, DW80, and DW100 at 25 °C, 10 °C, 0 °C, −10 °C, −20 °C, −30 °C, −40 °C, 25 °C, 40 °C, 50 °C, 60 °C. The two right figures are enlarged at −40 °C and 60 °C. (**b**) The cycling stability of three independent ZIHCs based on DW80 electrolyte at −40 °C at a current density of 0.5 A g^−1^. (**c**) The cycling stability of three independent ZIHCs based on DW80 electrolyte at 60 °C at a current density of 5 A g^−1^. The insert shows the mean± standard deviation of capacity retention and Coulombic efficiency.

The temperature-dependent electrochemical performance of ZIHCs assembled with DG-containing electrolytes was systematically evaluated at 1 A g^−1^ over a temperature range from −40 to 60 °C (Figure 6a). At room temperature (25 °C), all devices deliver comparable capacities, indicating that the incorporation of DG does not negatively affect the electrochemical behavior of ZIHCs. When the temperature decreases from 25 °C to −40 °C, all electrolytes experience different degrees of capacity reduction due to decreased ion mobility and increased electrolyte resistance. Among the investigated electrolytes, DW80 delivers the best low-temperature performance, maintaining a significantly higher reversible capacity at −40 °C compared with DW20, DW40, DW60, and DW100. In contrast, the capacity of DW20 decreases rapidly at subzero temperatures, while DW100 exhibits severe performance degradation with almost complete capacity loss at −40 °C. This result suggests that excessive DG incorporation does not necessarily improve low-temperature electrochemical behavior, likely due to increased electrolyte resistance and sluggish ion transport under low-temperature conditions.

When the temperature is gradually increased from −40 °C to 60 °C, all ZIHCs recover their electrochemical activity to different extents. The DW80 electrolyte maintains the highest reversible capacity and the most stable cycling behavior throughout the entire temperature range. At elevated temperatures, excessive water content in DW20 leads to accelerated capacity decay, while excessive DG addition in DW100 may cause unfavorable ion transport due to increased electrolyte viscosity. The stable CV responses of ZIHCs at 40 and 60 °C (Figures S7 and S8) further confirm the feasibility of DG-based electrolytes for high-temperature operation. In addition, the DW80 electrolyte exhibits reversible CV curves and charge/discharge profiles at different temperatures of −40, −20, 0, 25, 40, and 60 °C (Figure S9). These results demonstrate that an appropriate DG concentration is essential for achieving balanced wide-temperature electrochemical performance.

To further evaluate the long-term stability of the optimized electrolyte, the cycling performance of three independent ZIHCs assembled with DW80 was investigated under both low- and high-temperature conditions after a thermal equilibration of ≥30 min. As shown in Figure 6b, three DW80-based ZIHCs maintain stable cycling performance for 1713, 1682, and 1610 cycles at −40 °C with a current density of 0.5 A g^−1^, respectively. As summarized in the insert table, the ZIHC cells deliver an average capacity retention of 121.07 ± 0.83 and an average Coulombic efficiency of 99.87 ± 0.12. The stable low-temperature performance can be attributed to improved electrolyte stability and reduced water-related side reactions induced by DG incorporation, which facilitates Zn^2+^ transport and maintains reversible electrode reactions under low-temperature conditions.

The high-temperature cycling performance was also evaluated at 60 °C. As shown in Figure S10, the cycling stability of ZIHCs with different DG concentrations varies significantly, and the appropriate DG content contributes to improved capacity retention under elevated temperatures. This enhancement is mainly associated with the reduced water activity and the high boiling point of DG, which suppress excessive electrolyte degradation and zinc electrode corrosion. At a higher current density of 5 A g^−1^ (Figure 6c and Figure S11), the three DW80-based ZIHCs can operate continuously for 879, 848, and 825 cycles at 60 °C, respectively. As summarized in the insert table, the ZIHC cells deliver an average capacity retention of 80.83 ± 0.76 and an average Coulombic efficiency of 99.65 ± 0.12. The subsequent capacity decay is mainly related to the accumulation of parasitic reactions during prolonged high-temperature operation. Although the DW100 electrolyte exhibits comparable cycling stability at room temperature, excessive DG content introduces several limitations. The high-DG electrolyte is flammable, which compromises the safety advantage of aqueous electrolytes. Moreover, the DW100 electrolyte shows inferior electrochemical performance under subzero conditions, indicating that excessive DG addition cannot guarantee optimal low-temperature adaptability. Therefore, DW80 was selected as the optimized electrolyte because it provides a better balance among safety, temperature tolerance, and electrochemical stability. Furthermore, we compared our DW80 electrolyte with other widely used cosolvents (such as ethylene glycol (EG), acetonitrile (AN), dimethyl sulfoxide (DMSO), tetramethylurea (TMU), 1,1,2,2-tetrafluoroethyl-2,2,3,3-tetrafluoropropylether (TTE), dimethyl formamide (DMF), tetraglyme, and propylene carbonate (PC)) for wide-temperature aqueous zinc-ion systems (Table S5) [51,52,53,54,55,56,57]. The comparison demonstrates that DW80 combines a wide operating window (−40 to 60 °C) with non-flammability and high Coulombic efficiency, which is competitive with other cosolvents.

Overall, the wide-temperature evaluation demonstrates that the DG-based electrolyte, especially the DW80 composition, enables stable operation of aqueous ZIHCs across a broad temperature range from −40 to 60 °C. The improved performance originates from the synergistic effects of hydrogen bond interaction regulation, modification of the Zn^2+^ solvation environment, and suppression of water-related side reactions. This simple cosolvent strategy provides an effective approach for improving the temperature adaptability of aqueous zinc-based energy storage systems.

## 3. Materials and Methods

### 3.1. Materials

Diethylene glycol (DG) and zinc trifluoromethanesulfonate (Zn(OTf)_2_) were obtained from Aladdin Chemical Co., Ltd. (Shanghai, China). Deionized water was supplied by Macklin (Shanghai, China). Glass microfiber membranes (Whatman GF/D, Maidstone, UK) were employed as separators. Activated carbon (YP-80F, Kuraray, Tokyo, Japan), zinc foil (50 μm thickness), stainless steel mesh (SS), and copper foil (20 μm thickness) were purchased from Canrd (Dongguan, Guangdong, China). Before cell fabrication, zinc foils and stainless steel meshes were ultrasonically cleaned in a mixed ethanol/water solution (volume ratio of 1:1) to remove surface contaminants, followed by drying under ambient conditions.

### 3.2. Preparation of DG-Based Aqueous Electrolytes

Based on the Zn(OTf)_2_ concentration widely adopted in previously reported aqueous zinc-based electrolytes, 2 mol L^−1^ Zn(OTf)_2_ was selected as the baseline electrolyte concentration, and different amounts of DG were subsequently introduced to investigate the effect of DG content. Zn(OTf)_2_ was dissolved in deionized water to prepare the aqueous zinc electrolyte (2 mol L^−1^). Subsequently, different amounts of DG were introduced into the Zn(OTf)_2_ aqueous solution under continuous stirring until homogeneous electrolytes were obtained. The prepared electrolytes were denoted as DWx, where x represents the volume percentage of DG relative to water in the mixed solvent (e.g., DW20 corresponds to a DG/H_2_O volume ratio of 20:100). In this study, DW20, DW40, DW60, DW80, and DW100 (corresponding to DG/H_2_O volume ratios of 20:100, 40:100, 60:100, 80:100, and 100:100, respectively) were selected as the primary electrolytes for investigation. All electrolytes were continuously stirred until homogeneous solutions were obtained. Accordingly, the Zn(OTf)_2_ concentration was diluted in all electrolytes, as detailed in Table S1.

### 3.3. Materials Characterization

The ionic conductivity of different electrolytes at 25, 0, −20, and −40 °C was measured using a conductivity meter (DDSJ-380F, Leici, Shanghai, China). The viscosity of the electrolytes was measured using a rotational rheometer (MARS60, Thermo Scientific, Waltham, MA, USA) at 25 °C and −20 °C. Raman spectra were collected on a DXR3xi (Thermo Scientific, Waltham, MA, USA) spectrometer with a 532 nm laser at a spectral resolution of ≈2–4 cm^−1^; each spectrum was obtained from 10 accumulations of 20 s over the 500–4000 cm^−1^ range. The O–H stretching region (3000–3800 cm^−1^) was deconvoluted by nonlinear least-squares fitting with Gaussian components following the established two-component model for aqueous systems—strongly hydrogen-bonded water (~3230 cm^−1^) and weakly hydrogen-bonded water (~3430 cm^−1^). FTIR spectra were recorded on a Nicolet iS5 (Thermo Fisher Scientific, Waltham, MA, USA) in transmission mode at 4 cm^−1^ resolution with 10 scans. Each spectrum was baseline-corrected by polynomial subtraction prior to analysis.

The phase stability of different electrolytes at low and elevated temperatures was evaluated by visual observation under controlled temperature conditions. Differential scanning calorimetry (DSC) measurements were performed on a TA Instruments DSC25 (New Castle, DE, USA) equipped with a refrigerated cooling system. Approximately 5–10 mg of each electrolyte sample was hermetically sealed in an aluminum pan, and an empty sealed aluminum pan was used as the reference. The thermal program was set as follows: the sample was first equilibrated at 25 °C, then cooled from 25 °C to −80 °C at a ramp rate of 5 °C min^−1^, followed by heating from −80 °C back to 25 °C at the same rate of 5 °C min^−1^. The cooling and heating curves were recorded for analysis. The characteristic phase-transition temperatures (including freezing, melting, and glass transition temperatures) were determined from the DSC thermograms using the TA Instruments Trios software (VER 2.10.2.32).

The morphological evolution of Zn||Zn symmetric cells with different electrolytes during the Zn deposition process was observed using a YD650 optical microscope (YUESCOPE, Hefei, Anhui, China) in a homemade optical cell, where the current density and recording time were set to 10 mA cm^−2^, respectively. Morphological observations of the cycled zinc anode were performed using a field emission scanning electron microscope (FIB SEM GX4, Thermo Fisher Scientific). High- and low-temperature tests were conducted in a programmable environmental chamber (KW-Th-80s, Kowintest, Dongguan, Guangdong, China) covering a range from –50 to 80 °C.

### 3.4. Molecular Dynamics Simulations

Molecular dynamics (MD) simulations were performed to investigate the effects of DG incorporation on the electrolyte structure and Zn^2+^ solvation environment. All-atom MD simulations were performed using GROMACS 2023.3. The simulation systems were constructed based on the experimental electrolyte compositions. For the baseline 2 mol L^−1^ Zn(OTf)_2_ electrolyte, the system consisted of 60 Zn^2+^ ions, 120 OTf^−^ anions, and 1665 SPC/E water molecules in a cubic simulation box of L ≈ 3.68 nm (volume ≈ 50 nm^3^), corresponding to a salt concentration of ~2 mol L^−1^. For the DW80 electrolyte, the system consisted of 60 Zn^2+^ ions, 120 OTf^−^ anions, 1665 SPC/E water molecules, and 250 DG molecules in a cubic simulation box of L = 4.46967 nm (volume ≈ 89.4 nm^3^). The OPLS-AA force field was employed for all species. Energy minimization was performed using the steepest descent algorithm for 5000 steps with a time step of 1 fs. The system was then equilibrated in the NVT ensemble for 1 ns at 300 K using the Nose–Hoover thermostat, followed by NPT equilibration for 5 ns at 300 K and 1 bar. The production run was carried out in the NVT ensemble for 50 ns with a time step of 1 fs. Trajectory frames from 10 to 50 ns were used for structural analysis. The particle mesh Ewald (PME) method was used for long-range electrostatic interactions, with a real-space cutoff of 1.2 nm. Van der Waals interactions were treated with a cutoff of 1.2 nm and dispersion correction for energy and pressure.

### 3.5. Cell Assembly

All cells were assembled in an ambient atmosphere without inert gas protection. For Zn||Zn symmetric cells, both electrodes were zinc foil; for Zn||SS and Zn||Cu cells, zinc foil served as the anode, with stainless steel mesh and copper foil as the working electrodes, respectively. A glass microfiber separator was used in each cell, along with 100 μL of electrolyte. To prepare ZIHC cathodes, a mixture of activated carbon (YP 80F), acetylene black, and PTFE (8:1:1) was coated onto stainless steel mesh (electrode area of 1.13 cm^2^), yielding a mass loading of ∼1 mg.

### 3.6. Electrochemical Measurements

Electrochemical measurements, such as cyclic voltammetry (CV), galvanostatic charge–discharge (GCD), and electrochemical impedance spectroscopy (EIS), were performed using a CHI 660E electrochemical workstation (CHI Instruments, Shanghai, China). The cycling tests were run on a LAND system (CTA2001A, Wuhan, Hubei, China). The Tafel plots were measured using a Zn||Zn cell at a scan rate of 5 mV s^−1^. The corrosion currents and corrosion equilibrium potentials were automatically calculated by the CHI 660E software (Version 20.04). Hydrogen evolution and oxygen evolution activities were investigated using linear sweep voltammetry (LSV) at a scan rate of 5 mV s^−1^. The hydrogen evolution reaction (HER) and oxygen evolution reaction (OER) potentials were determined at an absolute current density of 10 mA. Zn||Cu asymmetric cells were assembled to evaluate zinc plating/stripping reversibility. Coulombic efficiency measurements were conducted under controlled current densities and areal capacities. Zn||Zn symmetric cells were assembled using zinc foil electrodes to investigate long-term cycling stability. The voltage profiles were recorded under constant current density. The voltage window of ZIHC cells was 0.2–1.8 V. The specific capacity (Q, mAh g^−1^) was calculated from the discharge profile as Q = j × Δt, where j is the current density (mA g^−1^, normalized to the total active material mass) and Δt is the discharge time (h). All high- and low-temperature electrochemical tests were performed after the cells were thermally equilibrated in the programmable environmental chamber for at least 10 min at the target temperature, to ensure that the electrolyte and electrodes reached a stable thermal state.

## 4. Conclusions

In summary, a diethylene glycol (DG)-based aqueous electrolyte was developed to enhance the wide-temperature performance of aqueous zinc-ion hybrid capacitors. The influence of DG concentration on electrolyte structure and electrochemical behavior was systematically evaluated. The results demonstrate that an appropriate DG content can effectively regulate hydrogen bond interactions among water molecules and modify the Zn^2+^ solvation environment, leading to reduced water-related side reactions and improved zinc plating/stripping reversibility. However, excessive DG addition does not continuously improve electrolyte performance, as the high DG content reduces the safety advantage of aqueous electrolytes and deteriorates low-temperature electrochemical behavior. Among the investigated compositions, the DW80 electrolyte provides an optimal balance between safety, temperature adaptability, and electrochemical stability. As a result, Zn||Zn symmetric cells and ZIHC devices assembled with the DW80 electrolyte exhibit stable cycling performance under both low-temperature (−40 °C) and elevated-temperature (60 °C) conditions. This study demonstrates that rational regulation of cosolvent content is essential for developing wide-temperature aqueous zinc-based energy storage systems.

## Data Availability

The data that support the findings of this study are available from the corresponding author upon reasonable request.

## Supplementary Materials

The following supporting information can be downloaded at: https://www.mdpi.com/article/10.3390/molecules31173001/s1, Table S1: Composition of the DG-based aqueous electrolytes; Table S2: DSC data of the DG-based aqueous electrolytes; Table S3: Ionic conductivity of the DG-based aqueous electrolytes; Table S4: Viscosity of the DG-based aqueous electrolytes at 25 and −20 °C; Table S5: Comparison of this work with previously reported wide-temperature aqueous zinc-based electrolytes using different cosolvents; Figure S1: Digital photographs of Zn(OTf)_2_-based electrolytes with different DG/H_2_O volume ratio in the mixed solvent under −55 °C; Figure S2: Deconvolution of the O–H stretching region into strongly hydrogen-bonded and weakly hydrogen-bonded water species in (a) DW20, (b) DW40, (c) DW60, and (d) DW80 electrolytes; Figure S3: FTIR spectra of pristine aqueous and DG-regulated electrolytes; Figure S4: Post-cycling SEM images of Zn electrodes from cycled three independent Zn||Cu cells. (a) 2 M Zn(OTf)_2_ electrolyte. (b) DW80 electrolyte; Figure S5: Zn||Zn symmetric cycling for the baseline 2 M Zn(OTf)_2_ and DW80 electrolytes at 1 mA cm^−2^ and 1 mAh cm^−2^ using three independent cells per electrolyte. Note that Zn||Zn cells based on DW80 electrolyte are still in continuous cycling; Figure S6: CV curves of ZIHCs assembled with different electrolytes at 25 °C; Figure S7: GCD curves of ZIHCs assembled with different electrolytes at 25 °C; Figure S8: Rate performance of ZIHCs assembled with different electrolytes at 25 °C; Figure S9: CV curves of ZIHCs assembled with different electrolytes at 40 °C; Figure S10: CV curves of ZIHCs assembled with different electrolytes at 60 °C; Figure S11: CV curves (50 mV s^−1^) of ZIHCs assembled with DW80 electrolytes at −40 °C, −20 °C, 0 °C, 25 °C, 40 °C and 60 °C; Figure S12: The cycling stability of ZIHC based on DW20, DW40, DW60, DW80 and DW100 electrolyte at 60 °C at low current density of 0.5 A g^−1^; Figure S13: The cycling stability of ZIHC based on DW20, DW40, DW60, DW80 and DW100 electrolyte at 60 °C at high current density of 5 A g^−1^.
