# Supplementary material for: A Diethylene Glycol-Regulated Aqueous Electrolyte for Zinc-Ion Hybrid Capacitors Operating from −40 to 60 °C
Source: Molecules. 2026 Aug 27;31(17):3001. doi: 10.3390/molecules31173001 (PMC13567059; doi:10.3390/molecules31173001)
Supplement: Supplementary file 1 [file molecules-31-03001-s001.zip › molecules-4498775-supplementary.pdf]

Supporting Information For

A Diethylene Glycol-Regulated Aqueous Electrolyte for Zinc-Ion Hybrid Capacitors Operating from −40 to 60 °C

Wentao Li, Ying Liu *, Xingchao Wang, Xiaohan Jiang, Lingyang Liu *

Shandong Provincial Key Laboratory of Chemical Energy Storage and Novel Cell Technology, School of Chemistry and Chemical Engineering & School of Energy Science and Technology, Liaocheng University, Liaocheng, 252059, China;

* Correspondence: liuy@lcu.edu.cn. (Y. Liu); liulingyang@lcu.edu.cn (L. Liu)

Table S1. Composition of the DG-based aqueous electrolytes.

Electrolytes	DG/H_2O volume ratio	DG vol%	DG/H_2O molar ratio	DG molar fraction	$Zn(OTf)_2$ concentration
$Zn(OTf)_2$	0:100	0	0.000:1	0	2.00 mol L^{-1}
DW20	20:100	16.7	0.038:1	3.66%	1.67 mol L^{-1}
DW40	40:100	28.6	0.076:1	7.06%	1.43 mol L^{-1}
DW60	60:100	37.5	0.114:1	10.23%	1.25 mol L^{-1}
DW80	80:100	44.4	0.152:1	13.19%	1.11 mol L^{-1}
DW100	100:100	50.0	0.190:1	15.97%	1.00 mol L^{-1}

Table S2. DSC data of the DG-based aqueous electrolytes.

Electrolytes	Cooling: Crystallization Tc (°C)	Heating: Melting Tm (°C)	Heating: ΔHm (J g^{-1})
$Zn(OTf)_2$	−65.2	−28.82	19.90
DW20	not detected	−27.75	31.69
DW40	not detected	−28.95	22.00
DW60	not detected	−32.26	13.09
DW80	not detected	not detected	~0.06
DW100	not detected	not detected	0.28

Table S3. Ionic conductivity of the DG-based aqueous electrolytes.

**Electrolytes**	**25 °C (ms cm^{-1}) (n=3)**	**0 °C (ms cm^{-1}) (n=3)**	**-20 °C (ms cm^{-1}) (n=3)**	**-40 °C (ms cm^{-1}) (n=3)**
$Zn(OTf)_2$	56.1 ± 0.10	42.4 ± 0.47	20.8 ± 0.62	Solidified
DW20	35.0 ± 0.26	22.7 ± 0.36	11.2 ± 0.30	1.34 ± 0.02
DW40	22.3 ± 0.06	11.9 ± 0.15	7.76 ± 0.05	0.617 ± 0.007
DW60	15.6 ± 0.15	11.0 ± 0.06	6.48 ± 0.02	0.439 ± 0.005
DW80	11.5 ± 0.15	7.50 ± 0.08	4.28 ± 0.03	0.223 ± 0.005
DW100	8.37 ± 0.03	5.20 ± 0.02	2.88 ± 0.02	0.113 ± 0.012

Table S4. Viscosity of the DG-based aqueous electrolytes at 25 and −20 °C.

**Electrolytes**	**Viscosity (mPa·s)**	
	**25 °C (n=3)**	**−20 °C (n=3)**
$Zn(OTf)_2$	3.37 ± 0.002	204.9 ± 1.2
DW20	6.30 ± 0.077	404.0 ± 0.45
DW40	6.31 ± 0.019	413.9 ± 0.43
DW60	6.80 ± 0.009	515.6 ± 0.72
DW80	10.81 ± 0.012	876.5 ± 2.30
DW100	24.13 ± 0.75	1420.5 ± 6.91

Table S5. Comparison of this work with previously reported wide-temperature aqueous zinc-based electrolytes using different cosolvents.

Cathode materials	Cosolvent	Electrolyte composition	Temperature Range (°C) and safety	Low-Temperature Cycling	High-Temperature Cycling	Refs.
Active Carbon	diethylene glycol (DG)	2 M $Zn(OTf)_2$ in H_2O/DG (100:80 by volume ratio)	–40 ~ 60 good safety	–40 °C: >1700 cycles (0.5 A g^{-1}) ~100% CE	60 °C: 850 cycles (0.5 A g^{-1}) ~99.8% CE	this work
Vanadium (III) oxide (V_2O_3)	Tetramethylurea (TMU)	2 M $Zn(OTf)_2$ in H_2O/ TMU (75:25 by volume ratio)	–50 ~ 90 good safety	–30 °C: 100 cycles (2 A g^{-1}) None CE	80 °C: 100 cycles (2 A g^{-1}) None CE	22
$VOPO_4 \cdot 2H_2O$	1,1,2,2-tetrafluoroethyl-2,2,3,3-tetrafluoropropylether (TTE)	0.83 m $Zn(TFSI)_2$ in H_2O–1.8 TFEA–2 TTE	–80 ~ 60 good safety	–30 °C: 5000 cycles (0.2 A g^{-1}) None CE	60 °C: 800 cycles (1 A g^{-1}) None CE	35
Polyaniline (PANI)	Ethylene Glycol (EG)	poly(2-acrylamido-2-methylpropanesulfonic acid)/polyacrylamide in EG/H_2O(10% v/v H_2O) containing $ZnCl_2$/NH_4Cl	–30 ~ 80 good safety	–30 °C: 1500 cycles (0.2 A g^{-1}) None CE	80 °C: 1000 cycles (5 A g^{-1}) None CE	51
PEDOT:PSS-PANI	Ethylene Glycol (EG)	1 M $ZnCl_2$ in EG/H_2O(40% volume ratio of EG to H_2O)	–30 ~ 40 good safety	–30 °C: 1000 cycles (2 A g^{-1}) None CE	40 °C: 1000 cycles (2 A g^{-1}) None CE	52
V_2O_5	Acetonitrile (AN)	1 M $Zn(OTf)_2$ in AN/H_2O (72 vol% AN)	–40 °C good safety	–40 °C: 1000 cycles (1 A g^{-1}) None CE	None	53
MnO_2	Dimethyl sulfoxide (DMSO)	2 M $ZnSO_4 \cdot 7H_2O$ in DMSO/H_2O (20 vol% DMSO)	–20 °C good safety	–20 °C: 300 cycles (1C) None CE	None	54
Active Carbon	Dimethyl Formamide (DMF)	2 mol L^{-1} $ZnSO_4$ in H_2O/DMF (H_2O:DMF = 4:2 by volume)	–30 ~ 70 good safety	–20 °C: 10000 cycles (0.5 A g^{-1}) None CE	60 °C: 1000 cycles (2 A g^{-1}) 70 °C: 600 cycles (2 A g^{-1}) None CE	55
Polyaniline (PANI)	Tetraglyme	2 M $Zn(OTf)_2$ with 88 mol% H_2O + 12 mol% Tetraglyme	–35 ~ 75 good safety	–35 °C: 1000 cycles (1 A g^{-1}) None CE	75 °C: 1000 cycles (5 A g^{-1}) None CE	56
Sodium vanadate ($Na_{0.33}V_2O_5$)	Propylene carbonate (PC)	2 m $Zn(ClO_4)_2$ in PC/H_2O = 6:4 (40 wt% WOE)	–20 ~ 50 good safety	–20 °C: 3000 cycles (2 A g^{-1}) None CE	50 °C: 100 cycles (2 A g^{-1}) None CE	57
Polyaniline (PANI)	Propylene carbonate (PC)	2 m $Zn(ClO_4)_2$ in PC/H_2O = 6:4 (40 wt% WOE)	–20 ~ 50 good safety	–20 °C: 1000 cycles (2 A g^{-1}) None CE	50 °C: 1000 cycles (2 A g^{-1}) None CE	57

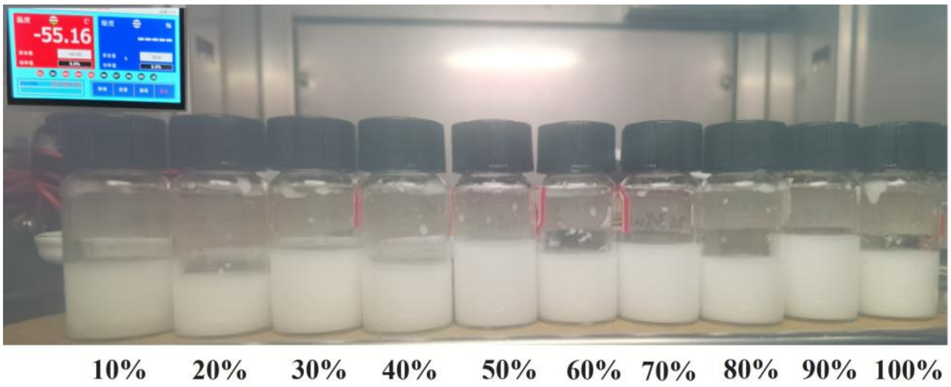

Figure S1. Digital photographs of $Zn(OTf)_2$-based electrolytes with different DG/H_2O volume ratio in the mixed solvent under −55 °C.

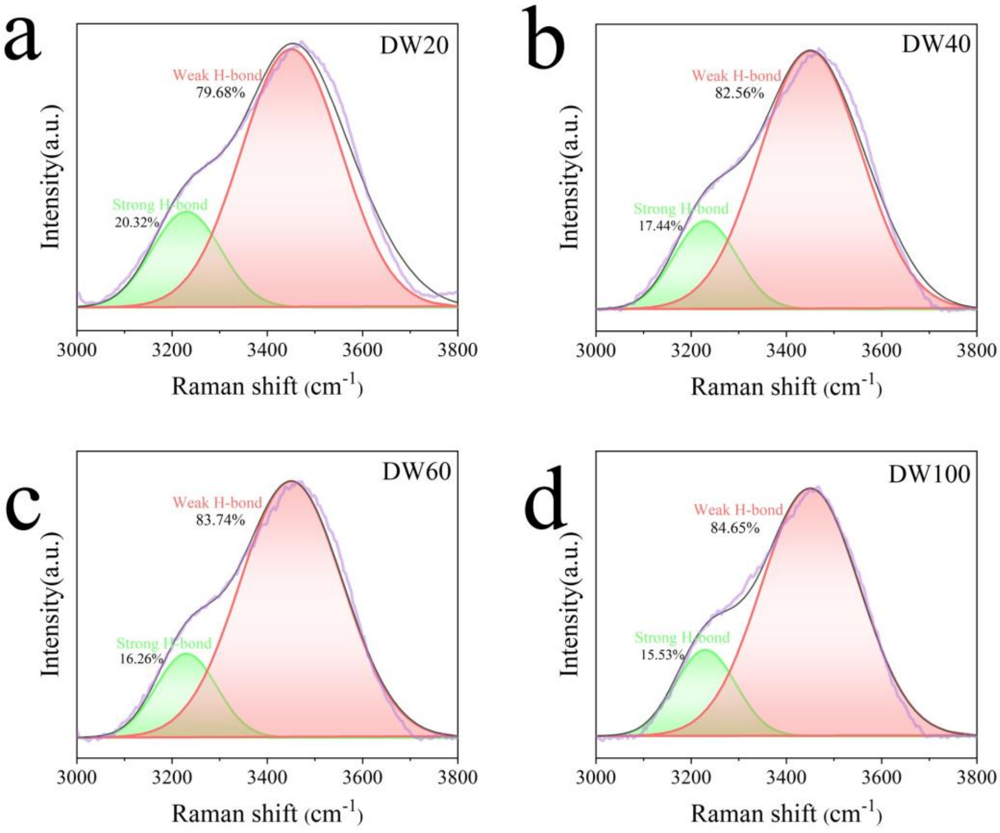

Figure S2. Deconvolution of the O–H stretching region into strongly hydrogen-bonded and weakly hydrogen-bonded water species in (a) DW20, (b) DW40, (c) DW60, and (d) DW80 electrolytes.

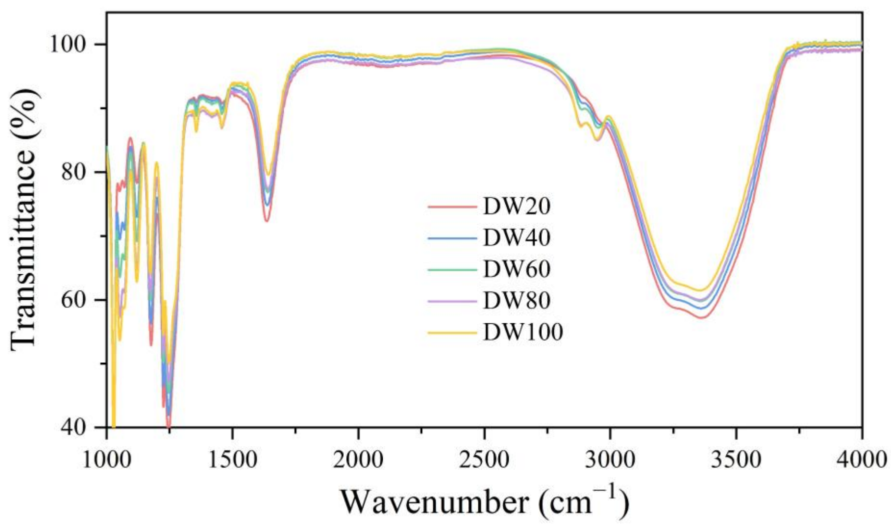

Figure S3. FTIR spectra of pristine aqueous and DG-regulated electrolytes.

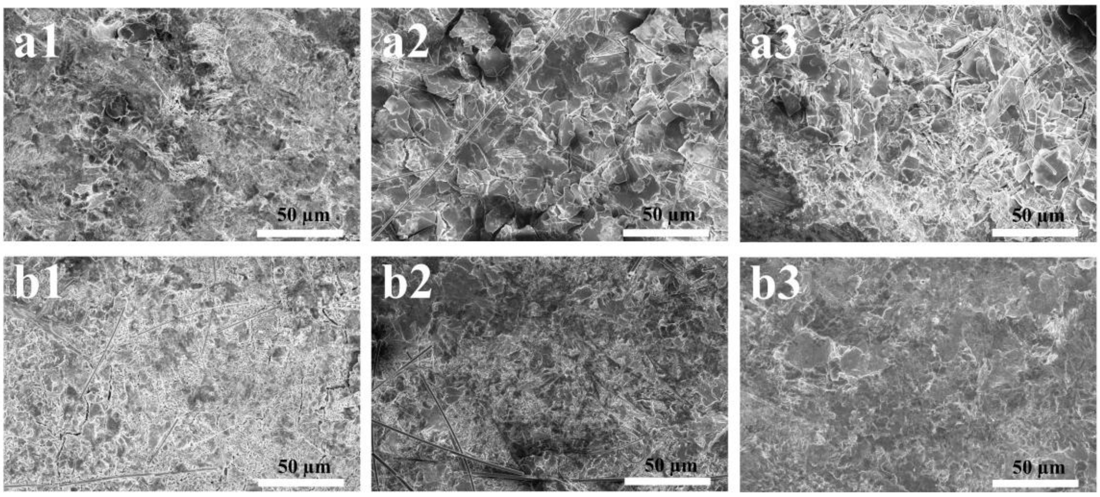

Figure S4. Post-cycling SEM images of Zn electrodes from cycled three independent Zn||Cu cells. (a) 2 M $Zn(OTf)_2$ electrolyte. (b) DW80 electrolyte.

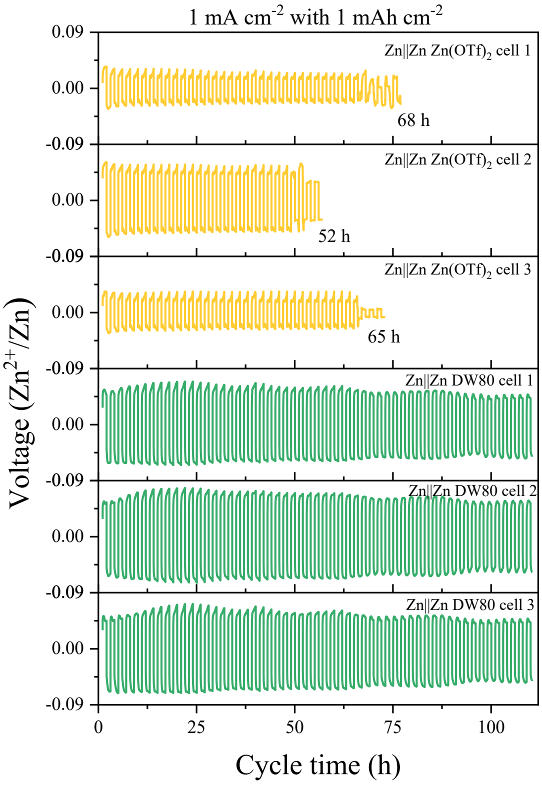

Figure S5. Zn||Zn symmetric cycling for the baseline 2 M $Zn(OTf)_2$ and DW80 electrolytes at 1 mA cm^{-2} and 1 mAh cm^{-2} using three independent cells per electrolyte. Note that Zn||Zn cells based on DW80 electrolyte are still in continuous cycling.

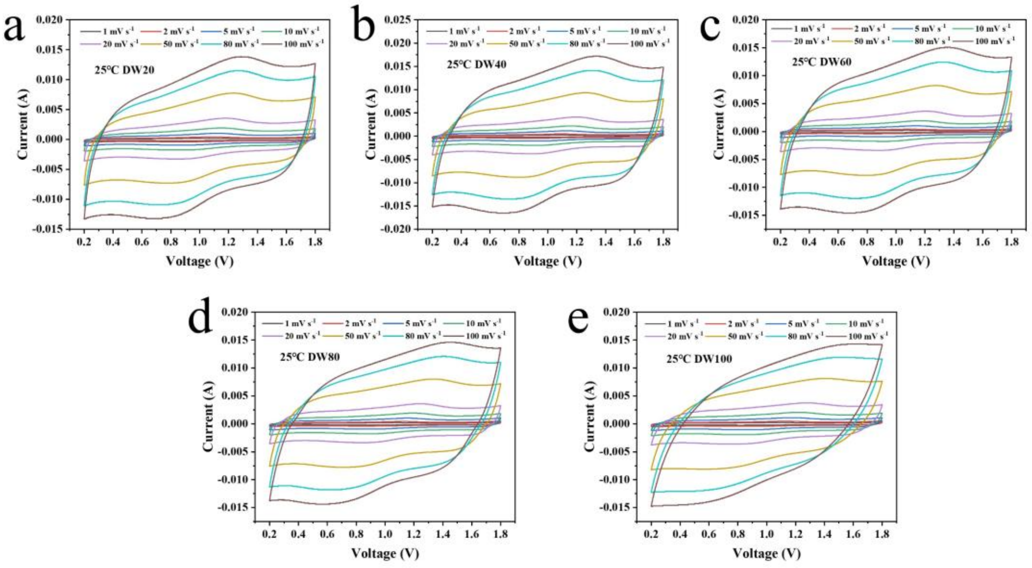

Figure S6. CV curves of ZIHCs assembled with different electrolytes at 25 °C.

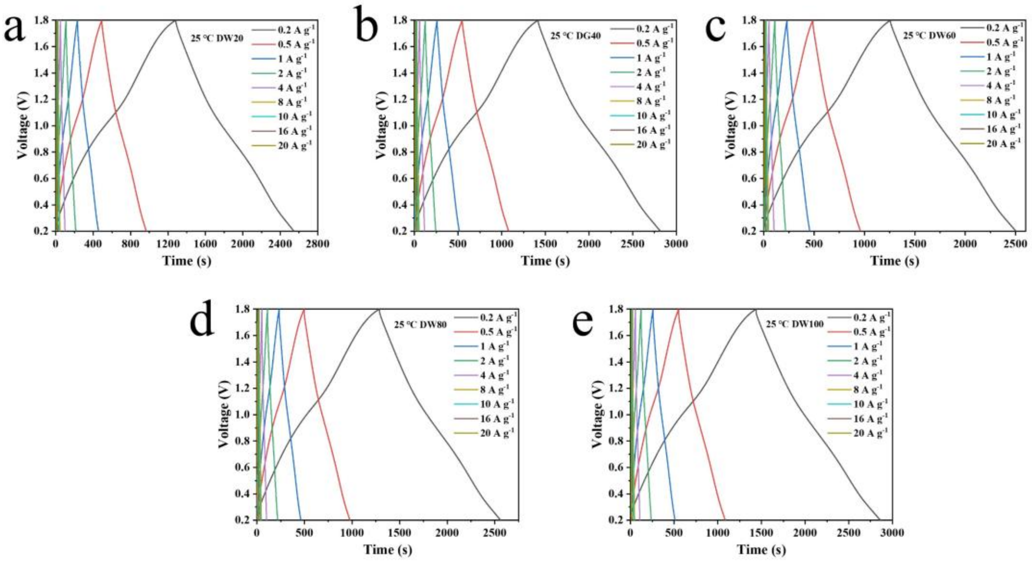

Figure S7. GCD curves of ZIHCs assembled with different electrolytes at 25 °C.

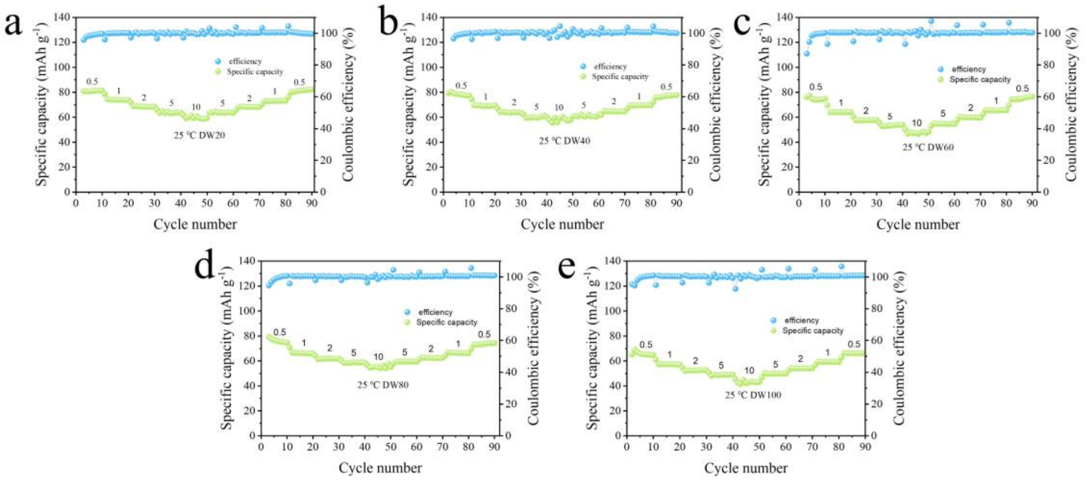

Figure S8. Rate performance of ZIHCs assembled with different electrolytes at 25 °C.

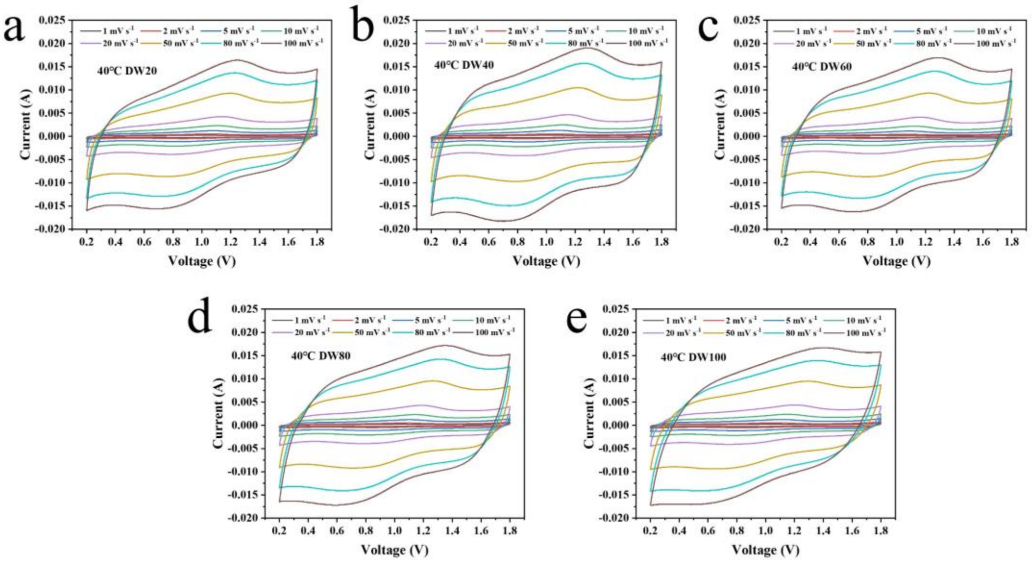

Figure S9. CV curves of ZIHCs assembled with different electrolytes at 40 °C.

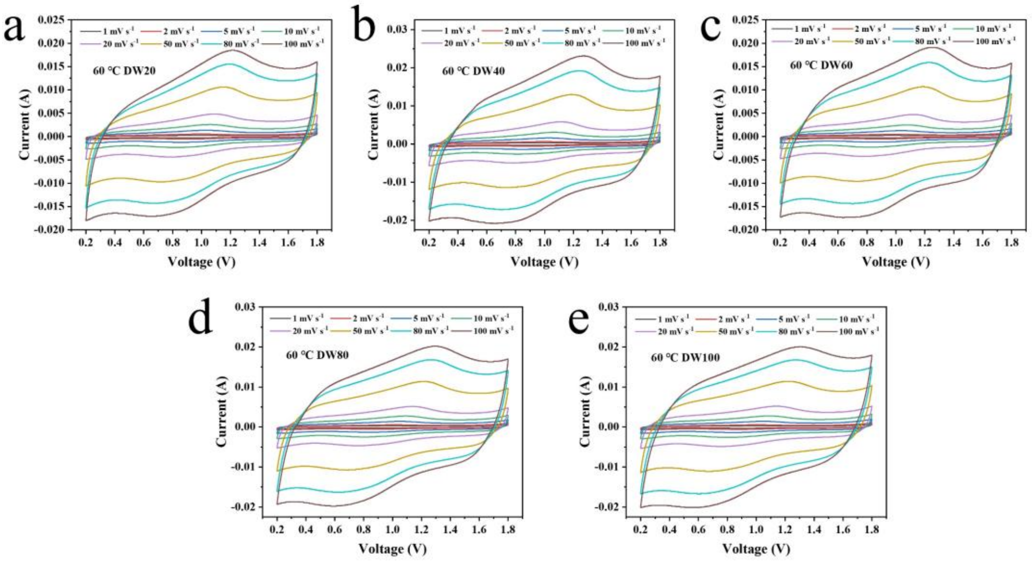

Figure S10. CV curves of ZIHCs assembled with different electrolytes at 60 °C.

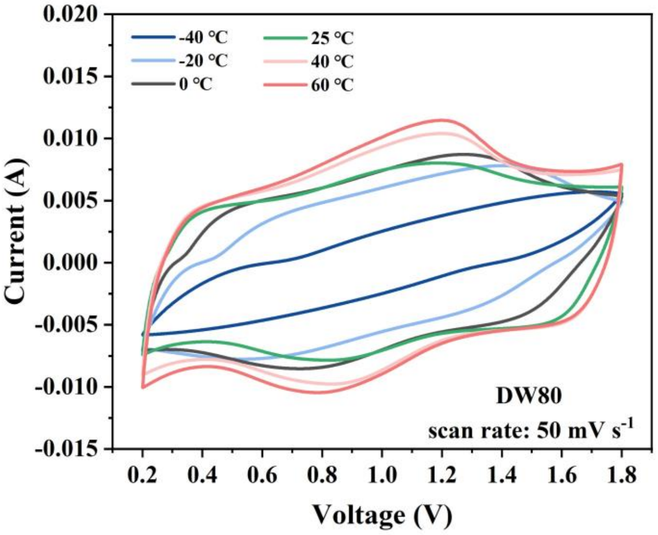

Figure S11. CV curves (50 mV s^{-1}) of ZIHCs assembled with DW80 electrolytes at -40 °C, -20 °C, 0 °C, 25 °C, 40 °C and 60 °C.

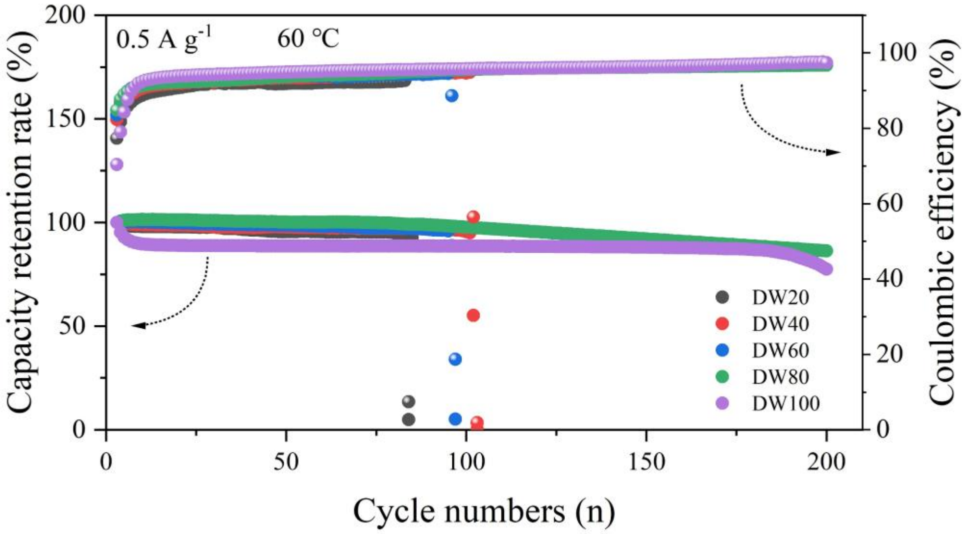

Figure S12. The cycling stability of ZIHC based on DW20, DW40, DW60, DW80 and DW100 electrolyte at 60 °C at low current density of 0.5 A g^{-1}.

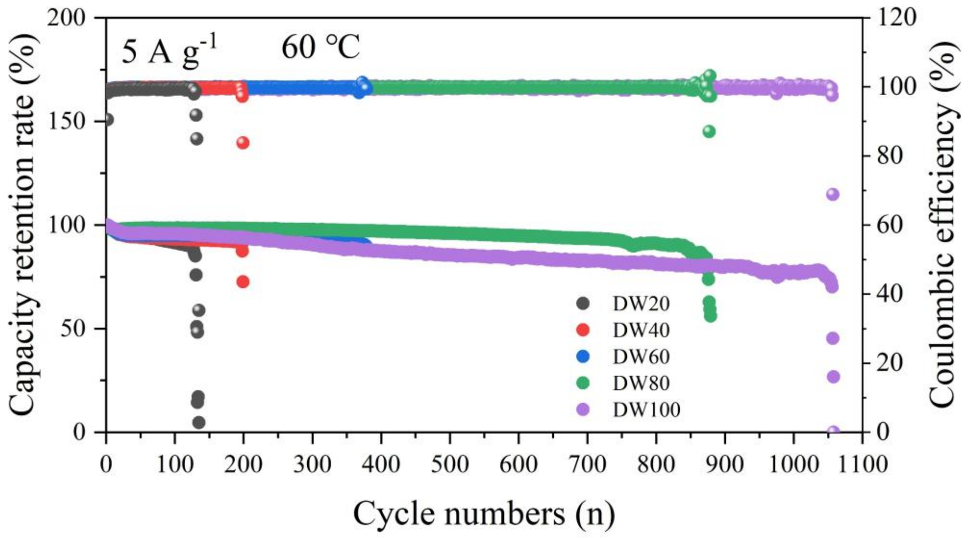

Figure S13. The cycling stability of ZIHC based on DW20, DW40, DW60, DW80 and DW100 electrolyte at 60 °C at high current density of 5 A g^{-1}.
